# Comparison of Craniofacial Anthropometric Measurement Accuracy of Manual Technique vs. Cone-Beam CT Scanning

**DOI:** 10.3390/diagnostics14222595

**Published:** 2024-11-19

**Authors:** Alexandru Misăiloaie, Ionuț Tărăboanță, Cristinel Ionel Stan, Cristian Constantin Budacu, Denisa-Mihaela Misăiloaie, Anca Sava

**Affiliations:** 1 Faculty of Dental Medicine, “Grigore T. Popa” University of Medicine and Pharmacy from Iasi, 16 Universitatii Str., 700115 Iasi, Romania; 2 “St. Spiridon” County Clinical Emergency Hospital, 700115 Iasi, Romania

**Keywords:** anthropometry, craniometry, infraorbital foramen, CBCT

## Abstract

Background: This study aimed to compare the accuracy of linear measurements obtained using the classical (manual) method versus cone-beam computed tomography (CBCT) in craniofacial anthropometry, specifically targeting the infraorbital foramen (IOF). Methods: This study involved two sample groups: one of 40 dry skulls measured manually using digital calipers, and the other of 40 CBCT 3D images digitally measured. Measurements included IOF height, distances between the IOF and nasion (N), IOF and frontomalar orbital (FMO), and between the two IOFs. Statistical analysis was performed using an ANOVA, t-test, and Fisher’s test with a significance level of 0.05. Results: The manual method recorded a mean IOF height of 2.1 mm, while CBCT showed a mean of 3.52 mm. Significant differences were recorded between the two methods when measuring IOF height, with a *p* < 0.001. However, CBCT measurements generally yielded higher accuracy and lower variance due to the absence of significant differences (*p* > 0.05). The distance between the two IOFs measured by both methods differed significantly (*p* = 0.03157), with CBCT measurements showing higher values. Conclusions: In conclusion, although no significant differences were found in the overall accuracy of the two methods, CBCT proved to be a more reliable tool for detailed craniofacial measurements due to its higher accuracy and reproducibility. CBCT demonstrated superior consistency in measurements, offering enhanced precision in assessing craniofacial structures. These findings support the growing preference for CBCT in maxillofacial surgery, where precision is critical for successful outcomes. Nonetheless, manual techniques remain valuable in settings where advanced imaging is not accessible.

## 1. Introduction

The morphological growth of the maxillofacial region is influenced by several factors, and understanding this phenomenon is crucial in medical specialties such as maxillofacial surgery [1]. Environmental or genetic factors, parafunctional habits, functional malocclusion, or muscle position and action are some of the elements that may influence the bone growth process [2,3].

First developed by Broadbent and Hofrath in 1931, craniofacial anthropometry is essential for measuring various craniofacial elements and is useful in the diagnosis, therapeutic planning, or evaluating the effectiveness of surgical treatment [4,5]. Measurements can be performed using various techniques, including direct measurement, photogrammetry, 3D laser scanning, computed tomography (CT), magnetic resonance imaging (MRI), 2/3-dimensional ultrasound, or cephalometric radiography [6]. 

The direct or traditional technique can be performed using various instruments such as the cranial compass, caliper, or millimeter ruler. This technique offers the advantage of reliability, low cost, and repeatability; however, it is time-consuming and laborious [7]. 

A more current and modern method used in craniofacial anthropometry is the computed tomography (CT) imaging technique. However, the use of CT as a diagnostic imaging method in this area is limited due to the high degree of irradiance and low resolution of the generated images. Nevertheless, cone-beam computed tomography (CBCT) has gained ground due to its short scanning time and the possibility of generating high-quality images with low radiation [1]. Furthermore, three-dimensional (3D) reconstructions, image transfer and archiving, real-time imaging, and 3D analysis of structures can be achieved by CBCT technology [8].

The application fields of the CBCT technique are various and include orthodontics, oral surgery, implantology, endodontics, and periodontics [1]. Data in the literature are contradictory, as some studies stated direct technique as the most accurate [1,9] while others showed that non-contact 3D imaging methods are more advantageous due to the possibility of accurate measurement of surfaces, angles, speed of image acquisition, and archiving [10].

A particularly important role in oral and maxillofacial surgery is played by the infraorbital nerve, which is the largest branch of the maxillary nerve [9]. It reaches the face through the infraorbital foramen (IOF) and has a role in sensory innervation of the lower eyelid, conjunctiva, upper lip, lateral surface of the nose and upper incisors, canines, and premolars [1,9]. Previous studies demonstrated the importance of anatomic landmarks, such as the infraorbital foramen (IOF), in surgical planning and in the prevention of complications [1,8]. The infraorbital foramen is a landmark of major importance in the infiltration of local anesthetics and in planning facial surgery. Its distances from other anatomic points, such as the nasion (N) and the frontomalar orbital (FMO) point, are of clinical interest. In the literature, measurements of this landmark have been performed both on dry skulls and with 3D imaging technologies such as CBCT [3,6].

However, few studies directly compare the accuracy of these two methods in the context of anthropometric measurements. The aim of this study was to compare the accuracy of linear measurements obtained by the classical technique on skulls with that obtained by CBCT imaging. The null hypothesis stated that no differences existed between the two measurement techniques and between the two halves of the face.

## 2. Materials and Methods

### 2.1. Study Design

The present study was conducted in accordance with the provisions of the Declaration of Helsinki and with the rules imposed by the Research Ethics Commission of “Grigore T. Popa” University of Medicine and Pharmacy of Iasi, Romania (ethical approval no. 186/17 May 2022).

The sample size was calculated using G*Power software version 3.1 (Heinrich-Heine Universität, Dusseldorf, Germany), with an effect size of 0,46, considered to be a high effect according to Cohen’s classification, an α value of 0.05, and a β value of 0.8. The obtained results indicated the use of a minimum total sample size of 40 samples.

**Figure 1:**
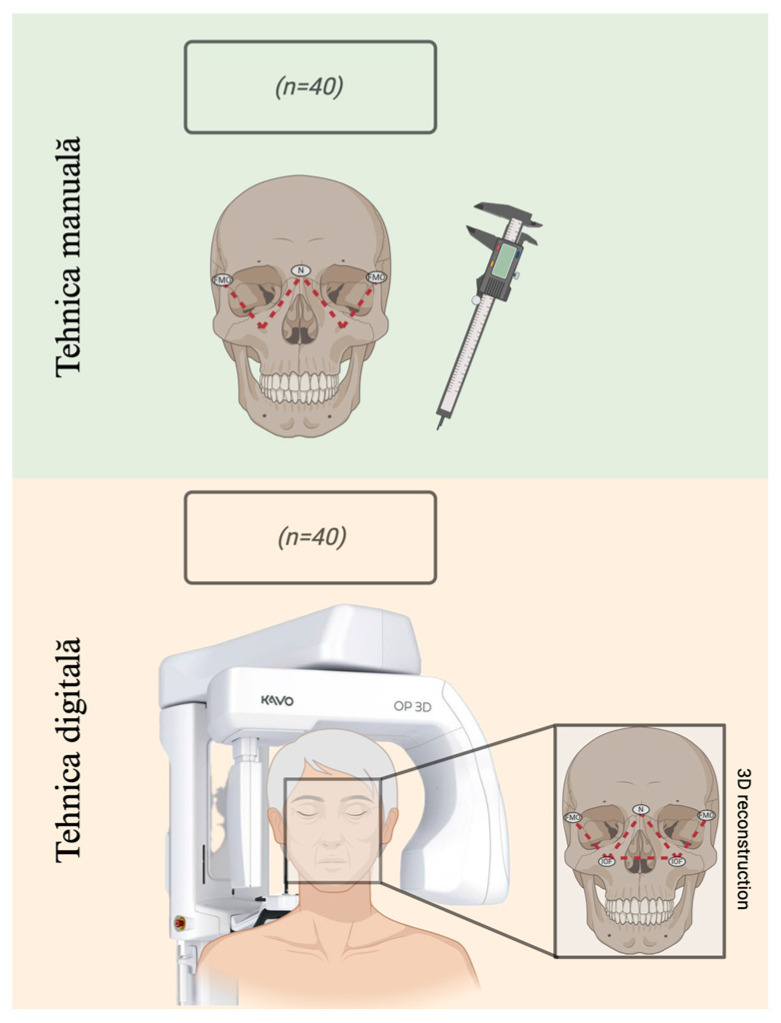
Study design.

This study included a total of 40 dry skulls selected from an osteologic collection from the Anatomy Department of “Grigore T. Popa” University of Medicine and Pharmacy of Iasi, Romania. The skull selection criteria were based on their structural integrity and the absence of post-mortem deformations. The study design is illustrated in Figure 1.

The measurements were performed directly on 40 dry skulls using a digital caliper. Each distance was measured two times to ensure the repeatability of the results. Two independent evaluators were involved in the measuring process in order to avoid intra-/inter-observatory variability.

The measurements on CBCT 3D images were performed using an ORTHOPANTOMOGRAPH OP 3D PRO (Kavo Dental, Biberach, Biberach, Germany) with a 6 cm Field of View, a Voxel of 0.2 mm, and a 40 s scanning time. A program compatible with the CBCT device, OnDemand 3D Project Viewer (Cybermed Company, Daejeon, Republic of Korea), was used to analyze the images. A total of 40 CBCT scans were involved in the study. The analysis of the CBCT images was performed by two investigators.

The following anthropometric points were considered:

⚬ Nasion (N)—the most anterior point located at the midline where the frontal and nasal bones intersect;
⚬ Infraorbital foramen (IOF)—the anterior opening of the infraorbital canal;
⚬ Frontomalar Orbital (FMO)—the frontomalar suture. The FMO point is located on the lateral orbital margin at the junction between the frontal and zygomatic bones.

Using both CBCT and manual techniques, the height of the IOF was measured. For the manual technique, the IOF height was measured by placing an extremity of the caliper on the superior margin of the infraorbital foramen and the other on the inferior orbital margin. The measurement was performed for both cranial halves. Similar techniques were used to measure the distances between the IOF and N points for both the right and left halves and the distances between the IOF and FMO.

### 2.2. Statistical Analysis

To assess the agreement between the values expressed by the two observers, the intra-class correlation coefficient (ICC) was calculated. For the assessment of the agreement between the two measurement methods for IOF height and the IOF-N, IOF-FMO, and IOF-IOF distances, we used the Bland–Altman test. To compare the groups, Fisher tests within a factorial ANOVA regression are versatile instruments, as the F-statistics provided are ratios that do not depend on the units the sample values are expressed in; therefore, there is no need to scale the samples in order to be able to compare them. Fisher tests give information if there is a factor that specifically influences the variance of the group and how much. Tukey tests offer additional information, providing values of the differences between samples. A *t*-test was also used in order to compare group means (Welch two samples test). For all the above tests, a significance threshold of *p* = 0.05 was established.

## 3. Results

### 3.1. IOF Height Determination

The height of the right infraorbital foramen for the 40 skulls measured using the classical method was 2.025 mm on average, and the left infraorbital foramen was 2.175 mm on average. The mean of the whole group was 2.1 mm.

**Figure 2:**
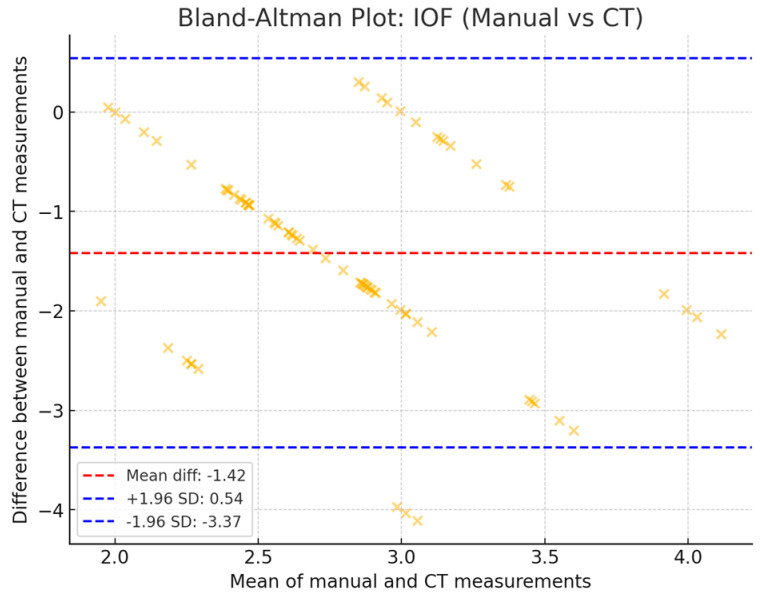
Bland–Altman test for the assessment of the agreement between the two measurement methods for IOF height (manual vs. CT).

The obtained ICC value of 0.734 indicates a moderate level of agreement between observers. The plot below (Figure 2) shows the difference between manual and CT measurements across the average values of both methods. The mean difference is small, and most points lie within the 95% limits of agreement.

Testing the variance of the foramen heights for the two sides of the skulls using the Fisher test and modeling with ANOVA factorial regression, the results were as follows: F-value = 1.418 and *p*-value = 0.237. The *p*-value was not significant (>0.05) and showed no significant difference between the two sides of the skull. The F-statistic of ~ 1 showed that there was homogeneity within the whole group of measurements (right and left) and that there were no relevant factors to differentiate the subgroups. 

**Figure 3:**
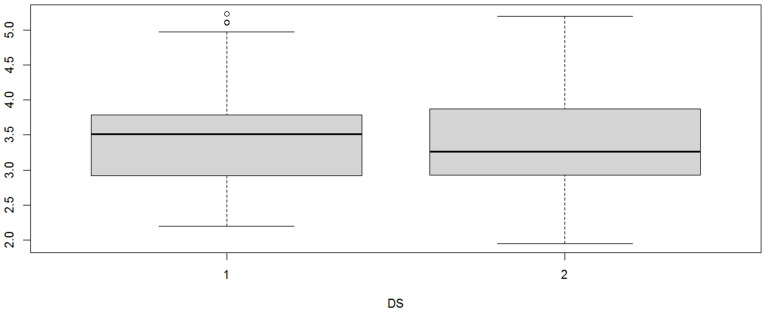
Mean values obtained by CBCT digital measurements of IOF height for right (1)/left (2) subgroups.

Although the *p*-value was not significant, the ANOVA model had a mean sum of squares of 0.4500, larger than the sum of residuals (0.3173), indicating that modeling the height of the infraorbital foramen (IOF) on every side of the skull (right/left) is correct; therefore, there are differences even if not significant. Using the CBCT method, the right IOF for the other 40 skulls was measured with a mean of 3.576 mm, and the left IOF had a mean of 3.458 mm. The mean of the whole group was 3.51675 mm (Figure 3).

**Figure 4:**
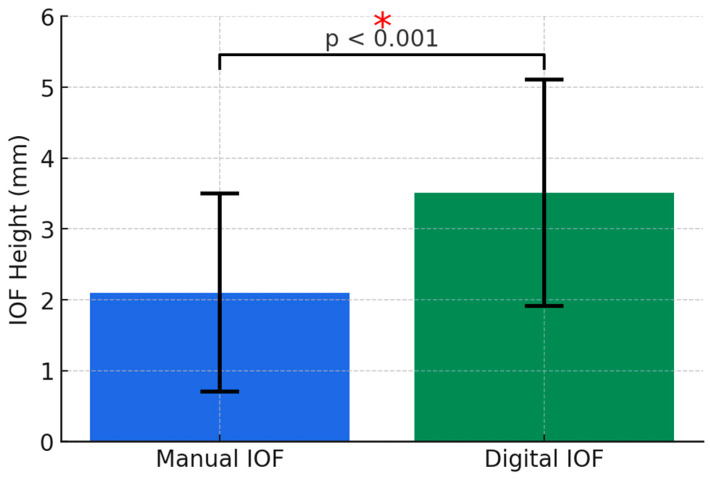
Mean values, standard deviations, and significance level of manual vs. digital measuring methods of IOF height. * Statistically significant differences.

Using the Fisher test again to observe the variance of the foramen heights on each of the two sides of the skulls and modeling with ANOVA factorial regression, the results were as follows: F = 0.434 and *p*-value = 0.512. The *p*-value (>0.05) was not statistically significant, and it showed there was no relevant difference between the two sides of the skulls. The F-statistic was very small and indicated that the variance of the whole group (right and left) was not very different from that of each side. The ANOVA model had a mean sum of squares of 0.2785, which was two times smaller than the sum of residuals, 0.6410. Therefore, such a model was not appropriate for explaining the variance of IOF height, meaning there were no relevant criteria to differentiate the sides of the skulls. The *p*-value was much higher than that of the subgroup measured by hand. The statistical analysis of the differences between the mean values obtained by the two groups (manual method vs. CBCT method) showed a *p*-value of <0.001. The mean values obtained from the digital measurements for the right/left subgroups are shown in Figure 4.

### 3.2. Measurements of the Distance Between IOF and the Nasion (N)

**Figure 5:**
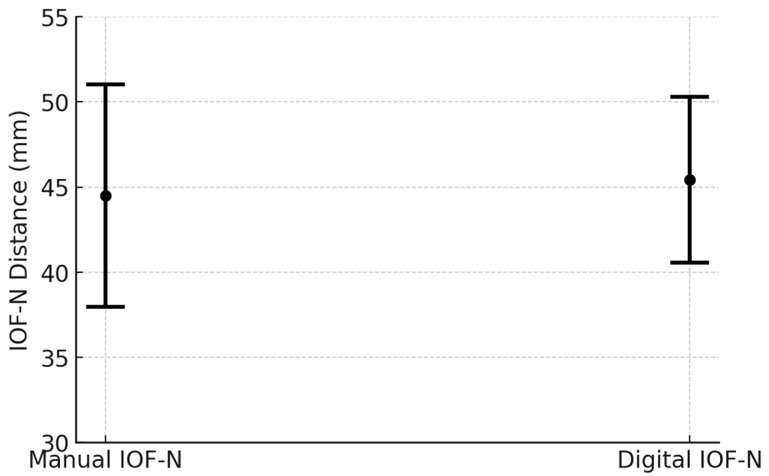
Comparison of IOF-N distances measured by manual and digital (CBCT) techniques.

Using the classical method, the distances had an average of 44.5 mm and an sd of 6.52551 mm. With the digital method, there was an average of 45.4142 mm and an sd of 4.873879 mm (Figure 5).

**Figure 6:**
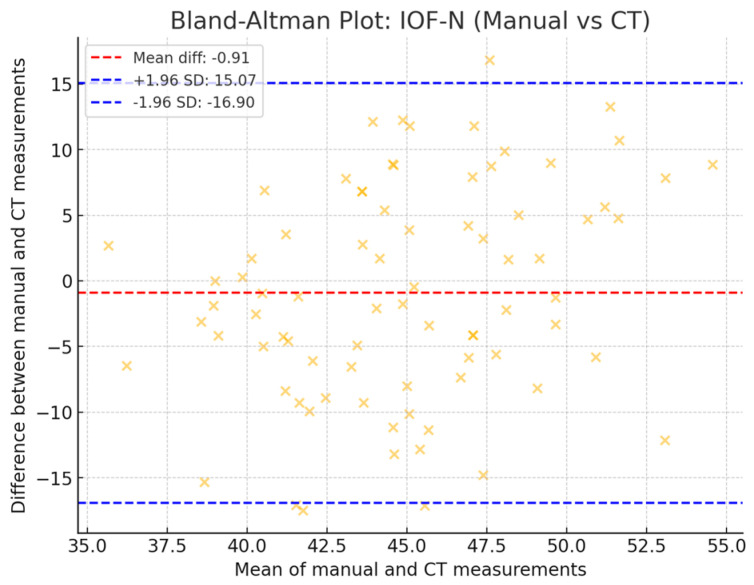
Bland–Altman test for the assessment of the agreement between the two measurement methods for IOF-N distance (manual vs. CT).

The agreement between the two evaluators was assessed using the intra-class correlation coefficient (ICC). The obtained ICC value of 0.721 indicated a medium level of consistency between the evaluators. Figure 6 illustrates the results of the Bland–Altman test for the assessment of the agreement between the two measurement methods for IOF-N distance (Manual vs. CT). This plot indicates a larger variability in the differences, but most data points still fall within the 95% limit.

Using ANOVA factorial regression on both sides of the skulls measured with the classical method, an F-statistic = 1.058 was obtained with the *p*-value = 0.307. The F-statistic had a value close to 1, indicating the variance of the whole group (right and left) was the same as in every subgroup (right/left). A *p*-value of > 0.05 was not significant, showing, similar to the F-statistic, that there was no factor in the subgroups to differentiate the distances no matter the skull side. 

On the other hand, the sum of squares in the regression model, 45.00, was slightly larger than the residuals, showing that it was appropriate to build the model on the skull sides since they differ. The average for the right side was 43.75 mm, and for the left, it was 45.25 mm.

With the digital method, modeling the sample with ANOVA factorial regression on both sides of the skulls, a very low value of F-statistic 0.003 was observed, with a very high *p*-value of 0.96, indicating that choosing the sides of the skull as a differentiating factor within the digital group is not effective. Observing the subgroup means, they indeed had very close values: 45.39 mm (right) and 45.44 mm (left).

### 3.3. Measurements of the Distance Between the Infraorbital Foramen (IOF) and the Frontomalareorbitale Point (FMO)

**Figure 7:**
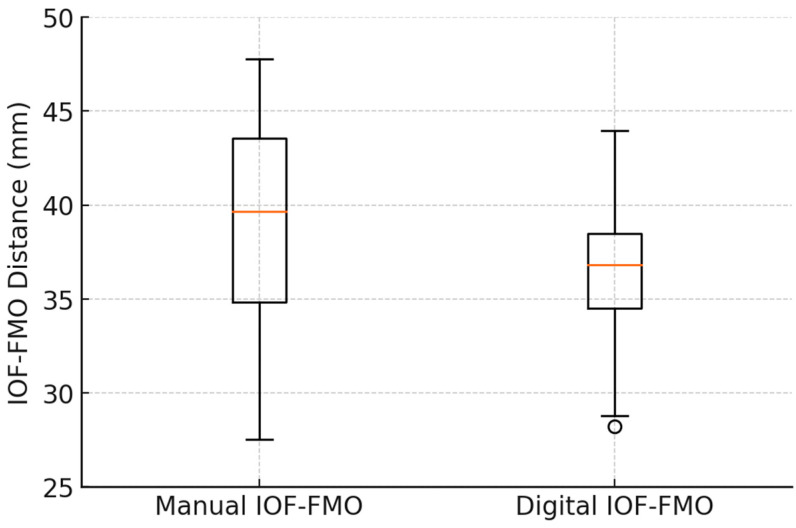
Comparison of IOF-FMO distances measured by manual and digital techniques.

With the classical method, the IOF-FMO distance had a mean of 37.95 mm and an sd of 4.157957 mm. Using the digital method, there was an average of 36.26263 mm and an sd of 3.038918 mm (Figure 7).

**Figure 8:**
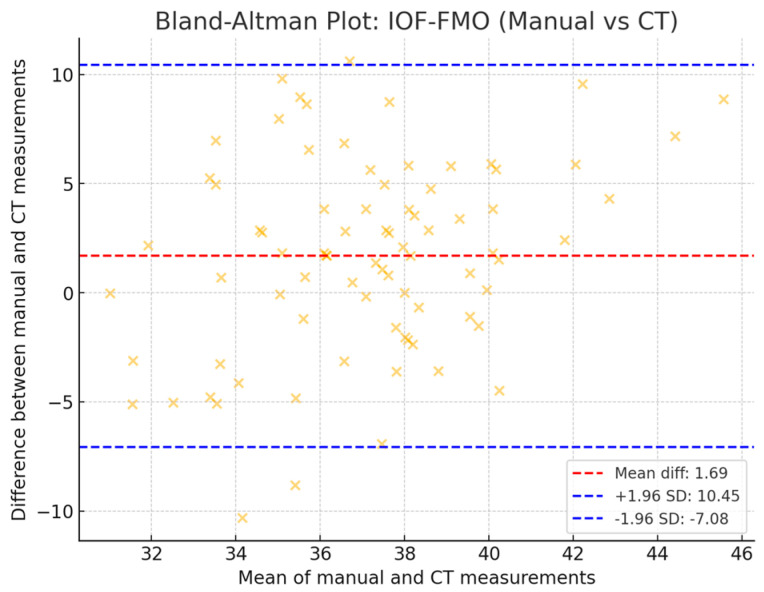
Bland–Altman test for the assessment of the agreement between the two measurement methods for IOF-FMO distance (manual vs. CT).

For the evaluation of the agreement between the values obtained by each measuring method, the Bland–Altman test was used (Figure 8). The results showed that the differences were distributed symmetrically around the mean difference, and most points were placed within the 95% limits of agreement. 

The results of the ANOVA factorial regression on both sides of the skulls measured manually were an F-statistic of 0.026 with a *p*-value of 0.873. Since the F-value was small and the *p*-value was high, there was no significant variance between the two subgroups. 

The average distance for the right side was 37.87 mm, and for the left side, it was 38.02 mm. 

For the digital method, the results of the ANOVA factorial regression on both sides of the skulls were an F-value of 0.679, not very high but larger than the classical method, with a *p*-value of 0.412, a high value but lower than the classical method. The IOF-FMO distances were more differentiated than previously. Checking the average values, the right side had an average of 35.98 mm, and the left side had an average of 36.54 mm.

The mean IOF-FMO distance was 0.56125 mm, which was larger than the classical method (0.15 mm). Since there was a better *p*-value and F-statistic, it was not an influence of the digitally measured skulls being bigger. On the contrary, the average (36.26263 m) was lower than the classical ones (37.95 mm). In fact, most of the CBCT distances were lower.

### 3.4. Measurements of the Distance Between the Two Infraorbital Foramens (IOF-IOF)

The distances between the two foramens for the subgroup measured by hand had an average of 49.4 mm, CI 95% [32.0, 71.0], and for the digitally measured one, an average of 52.64 mm, CI 95% [46.07, 62.81]. 

**Figure 9:**
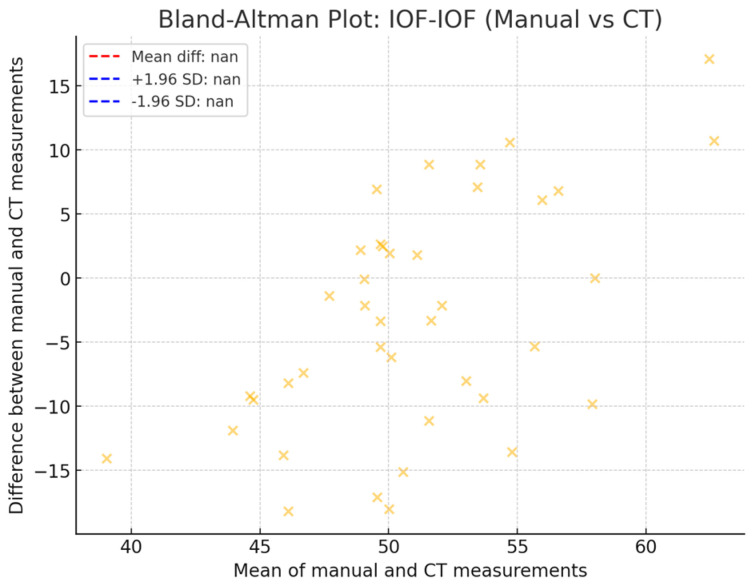
Bland–Altman test for the assessment of the agreement between the two measurement methods for IOF-IOF distance (manual vs. CT).

Figure 9 illustrates the results of the Bland–Altman test for the assessment of the agreement between the two measurement methods for IOF-IOF distance (manual vs. CT). This measurement also showed a strong agreement between the two methods. The mean difference was small, and nearly all points were within the 95% confidence range.

**Figure 10:**
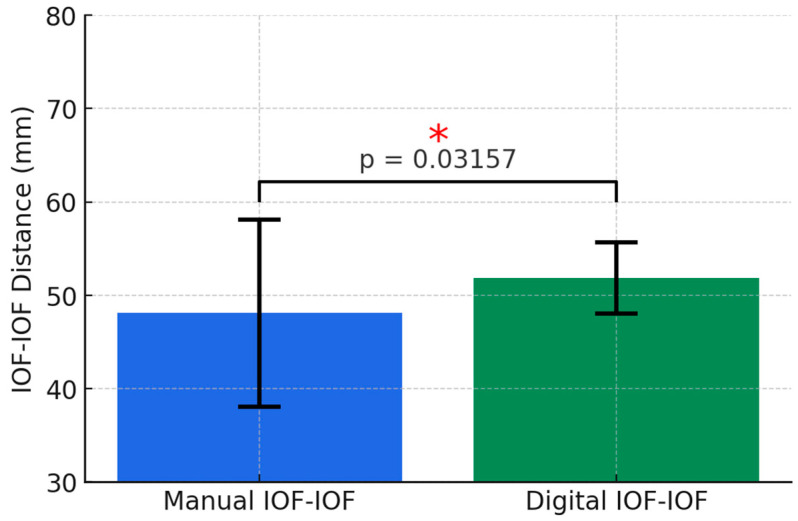
Comparison of IOF-IOF distances measured by manual and digital (CBCT) techniques. * Statistically significant differences.

The obtained ICC value of 0.847 indicated a high level of agreement between observers. Running the t-test (Welch Two Sample *t*-test), there was a difference between subgroup means *t* = −2.2016, *p*-value = 0.03157, and 95% confidence interval: [−6.1779225, −0.2955775] (Figure 10).

## 4. Discussion

The main objective of this study was to analyze the morphometry of the infraorbital foramen using the classical/manual technique of craniofacial anthropometry and CT scanning and to compare the accuracy of the two techniques. These analytical tools allow easy identification of the position of the infraorbital foramen and are easily reproducible. However, it is necessary that the measurement techniques be precise and accurate in expressing the dimensions given the imminence of intra- or interobserver errors [5,11].

Anthropometry is described as an easy-to-perform, non-invasive, low-cost technique that can be applied in various epidemiologic and clinical settings [12]. The purpose of this technique is to collect information on the regional or total dimensions of the human body using instruments such as rulers, calipers, manometers, scales, or skinfold calipers [12,13]. The word originated from a combination of the Greek terms anthropos (human) and metron (measure) and represents a method of measuring volumes, areas, or segments of the human body [14]. 

Anthropometry arose from the need to study the shape of the human body and its variability and was of particular importance in assessing nutritional status during the First World War. A limitation of this technique is that its accuracy depends on the skill of the operator and the patient’s training [12]. 

In recent years, it has been proposed to shift from two-dimensional to three-dimensional anthropometric analysis, so the use of CBCT has increased significantly. Three-dimensional images are much more accurate and reliable and allow a detailed assessment of all structures [15,16]. Despite the numerous advantages of CBCT, its accessibility remains limited for some patients; therefore, in such situations, the classical/manual technique for craniofacial anthropometry is the only option. For this reason, in the present study, we aimed to evaluate the degree of accuracy and correlation of measurements generated by the manual vs. digital CBCT technique. 

Our research was performed on a sample group of 40 dry skulls and a group of 40 CBCT 3D reconstructions. Previous studies showed similarities between the results obtained by the two techniques following the measurement of the infraorbital foramen height [17,18]. These results are in agreement with the results obtained in our study in which the mean values obtained following manual vs. CBCT scanning measurements of IOF height did not significantly differ between the two techniques, neither for the total mean values recorded for all the evaluated IOF nor for the half-skull analysis. Likewise, by comparing the dimensions of 40 skulls measured using the classical ruler and digital caliper method with those of another 40 skulls digitally measured on CBCT 3D reconstruction using Fisher’s statistical tests, ANOVA factorial regressions, the F-statistic, and *p*-values, the resulting significance levels were not statistically significant. No significant differences were determined between skull sides in either sample. Our results are in agreement with the findings of a study in which the comparison of the dimensions between the two skull halves showed no statistically significant differences [19,20]. 

However, several studies have indicated significant diversity in the shape and positioning of the IOF in different populations and ethnic groups, implying potential challenges for maxillofacial surgeons [21,22,23]. It is also important to emphasize the possibility of the existence of an accessory infraorbital foramen, which may increase the complexity of this region and require a thorough understanding of the relative position of the IOF.

In our study, statistical indicators showed lower levels for the F-statistic and higher *p*-values for the CBCT-measured sample, indicating higher homogeneity than the sample measured by the classical technique and smaller differences between the following hemisphere distances (left/right): height of the infraorbital foramen and for the IOF-nasion distances. Mean values of the measured dimensions were also higher in the CBCT-measured sample. The IOF-FMO distances were smaller in the CBCT subgroup and had larger differences between the two halves of the skull than in the manually measured group. 

For the IOF-IOF distances, there were significant differences between the means of the two samples, indicating higher values for the CBCT subgroup. By testing the normality of residuals of ANOVA models, the constancy of variance of the residuals, and outlier analysis, the conclusions were that CBCT measurements provide results much more faithful to observations and quantifiable data. The findings of our study are in agreement with the results of other studies showing that three-dimensional imaging techniques benefit from very high accuracy compared with direct or two-dimensional techniques [5,24,25,26].

Anthropometric analysis is crucial in the field of maxillofacial surgery in order to know the precise localization of the IOF to ensure an effective infraorbital nerve block and prevent any infraorbital nerve injury during mid-facial surgery such as orthognathic surgery, aesthetic surgery, and treatment of mid-facial fractures [7,12].

With the evolution of cone-beam computed tomography for the maxillofacial area, oral surgeons can evaluate the region using numerous imaging planes [27,28]. Cone-beam computed tomography can provide multiplanar images, such as axial, sagittal, and coronal views. Therefore, many researchers are now choosing three-dimensional (3D) CBCT analysis to assess maxillomandibular morphology, which offers better image resolution, shorter time, lower radiation dose, and lower cost [13,28,29]. The obtained results prove the advantages of using the CBCT method compared to direct measurements on dry skulls. CBCT provides superior accuracy, especially for measurements of depth and smaller distances, which can be difficult to achieve with manual instruments. This is in line with previous studies that emphasized the advantages of imaging technology in anthropometric measurements [11,26]. One of the main advantages of the CBCT technique is the possibility of obtaining three-dimensional images that reduce the errors associated with two-dimensional measurements on dry skulls. On the other hand, direct measurements on dry skulls remain an affordable and relatively accurate method and are widely used in situations where access to CBCT technology is limited [26].

The limitations of this study refer to the small sample size and the fact that only dry skulls were used, which do not perfectly reflect lifetime conditions. Measurements on dry skulls only provide information about bone structure without considering the presence of soft tissues that may influence height and anatomical dimensions during life. Soft tissues can alter the apparent position of some anatomical landmarks, which means that measurements on dry skulls are not directly applicable to living patients. In addition, even when using high-precision measuring instruments, manual measurements on skulls can be susceptible to human error. Incorrect tilting of the caliper or inaccurate placement of reference points can increase the variability of the data. Even if multiple investigators participated in the measurements, there may be interobserver variability in how anatomic landmarks are identified and measured, both in direct measurements on dry skulls and on CBCT images.

A major limitation of the present study was the absence of strict inclusion and exclusion criteria for the studied subjects or the analyzed dry skulls based on age, ethnicity, or race. Therefore, there was no demographic division of the subjects or skulls when included in the study. Also, the limited number of observers and the small amount of craniometric landmarks analyzed could limit the study’s relevance. Future studies should also include samples of living patients to compare measurements from CBCT images with those from dry skulls. Also, studies could involve larger samples of skulls from diverse populations and ethnic groups to ensure better representativeness and generalizability of results. The inclusion of a larger number of living patients could help to correlate measurements made on dry skulls with those on CBCT images. Moreover, future research could address how craniofacial anatomical landmarks change over time by performing repeated measurements on the same patients to observe how the structures change with age or following surgery.

## 5. Conclusions

- The analysis of the values resulting from the measurements at each half of the face showed the absence of significant differences for both tested craniofacial anthropometry methods;
- The CBCT imaging method showed a significantly larger dimension of the distance between the right and left infraorbital foramen compared to the manual method;
- By analyzing the variance consistency of residuals and outlier analysis, it can be concluded that CBCT measurements provide results more faithful to observations and quantifiable data;
- These findings support the growing preference for CBCT in maxillofacial surgery, where precision is critical for successful outcomes. Nonetheless, manual techniques remain valuable in settings where advanced imaging is not accessible.

## Data Availability

The original contributions presented in the study are included in the article; further inquiries can be directed to the corresponding author.
